# Prognostic Potential of Tumor-Infiltrating Immune Cells in Resectable Oral Squamous Cell Carcinoma

**DOI:** 10.3390/cancers13092268

**Published:** 2021-05-08

**Authors:** Ana Caruntu, Liliana Moraru, Mihai Lupu, Florina Vasilescu, Marius Dumitrescu, Mirela Cioplea, Cristiana Popp, Alexandra Dragusin, Constantin Caruntu, Sabina Zurac

**Affiliations:** 1 Department of Oral and Maxillofacial Surgery, “Carol Davila” Central Military Emergency Hospital, 010825 Bucharest, Romania; ana.caruntu@gmail.com (A.C.); liliana.moraru@yahoo.com (L.M.); 2 Department of Oral and Maxillofacial Surgery, Faculty of Dental Medicine, “Titu Maiorescu” University, 031593 Bucharest, Romania; 3 Dermatology Research Laboratory, “Carol Davila” University of Medicine and Pharmacy, 050474 Bucharest, Romania; lupu.g.mihai@gmail.com; 4 Department of Pathology, “Carol Davila” Central Military Emergency Hospital, 010825 Bucharest, Romania; florinapath@yahoo.com (F.V.); marius_med@yahoo.com (M.D.); 5 Department of Pathology, “Carol Davila” University of Medicine and Pharmacy, 020125 Bucharest, Romania; mirelacioplea@yahoo.com (M.C.); dragusin_alexandra88@yahoo.com (A.D.); sabina_zurac@yahoo.com (S.Z.); 6 Department of Pathology, Colentina University Hospital, 020125 Bucharest, Romania; brigaela@yahoo.com; 7 Department of Physiology, “Carol Davila” University of Medicine and Pharmacy, 050474 Bucharest, Romania; 8 Department of Dermatology, “Prof. N.C. Paulescu” National Institute of Diabetes, Nutrition and Metabolic Diseases, 011233 Bucharest, Romania

**Keywords:** head and neck cancer, oral squamous cell carcinoma, immunohistochemistry, tumor-infiltrating immune cells, lymphocytes, prognostic

## Abstract

**Simple Summary:**

Oral cancer is a common malignancy with high morbidity and mortality. Tumor-infiltrating immune cells play key roles in its pathogenesis, influencing tumor behavior and evolution. The aim of our study was to assess the prognostic character of tumor-infiltrating CD4^+^, CD8^+^ and CD56^+^ cells in oral squamous cell carcinoma. We found major differences in CD4^+^ and CD8^+^ lymphocyte density at the front of invasion compared to the intratumor compartment. In addition, intense infiltration with CD8^+^ lymphocytes in both compartments carried a positive prognostic character. Moreover, we found that a higher intratumor infiltration with CD56^+^ cells significantly correlated with locoregional disease control and improved survival. Our study confirms the key role of innate and adaptive immune systems in oral squamous cell carcinoma. The predictive characteristics of CD8^+^ and CD56^+^ cells can be implemented as independent prognostic tools and can provide important elements in developing individualized therapy in the fight against oral cancer.

**Abstract:**

(1) *Background*: The immune microenvironment plays an important role in carcinogenesis and has prognostic potential in many types of cancer. In this study we assess the prognostic character of tumor-infiltrating immune cells CD4^+^, CD8^+^ and CD56^+^ in resectable oral squamous cell carcinoma (OSCC); (2) *Methods*: We have evaluated the densities of CD4^+^, CD8^+^ and CD56^+^ in two distinct compartments, intratumor and invasion front, in 90 patients with OSCC; (3) *Results*: Significant differences were found between the tumor compartments for the CD4^+^ and CD8^+^ lymphocytes. An improved outcome (OS) was seen in patients with high densities of intratumor CD8^+^ lymphocytes (*p* = 0.0086), CD8^+^ lymphocytes at the front of invasion (*p* = 0.0011) and for intratumor CD56^+^ cells (*p* = 0.0016). Multivariate analysis confirmed the independent prognostic role of CD8^+^ at the front of invasion (OR = 3.75, CI95% 1.17–12.35, *p* = 0.026) and for intratumor CD56^+^ cells (OR = 3.669, CI95% 1.09–15.37, *p* = 0.035); (4) *Conclusions*: Tumor-infiltrating CD8^+^ lymphocytes at the front of invasion and CD56^+^ in the intratumor compartment display predictive traits in OSCC. A reach immune infiltration with these types of cells is associated with an improved patient outcome.

## 1. Introduction

Oral squamous cell carcinoma (OSCC) is included in the wider group of head and neck squamous cell carcinoma (HNSCC), which is the eighth most common cancer in the world [1]. This epithelial cancer arises from the mucosal layer of oral anatomical subsites: labial, buccal, gingival, lingual, floor of the mouth and palate mucosa. The main risk factors for OSCC are tobacco and alcohol abuse, alongside chronic exposure to ultraviolet (UV) radiation, malnutrition, poor oral hygiene and constitutional characteristics: age and gender [2,3]. OSCC can be easily detected by a simple examination allowing the identification of suspect lesions affecting oral mucosa. When detected early and treated, survival in OSCC reaches 90%, while in advanced disease, survival drops below 50% [4]. Despite that, almost half of the patients have advanced disease at first diagnosis that affects not only survival but also their quality of life [5,6,7]. Natural disease progression to advanced stages and therapeutic sequelae have severe aesthetic and functional consequences, with impairment of speech, swallowing and a major social impact on patients [8]. The standard of treatment in OSCC includes surgery, radiotherapy and chemotherapy in different combinations [9]. Recently, immunotherapy has been added to the therapeutic arsenal in patients with advanced and metastatic disease [10,11]. The immune system is one of the leading actors in the pathogenesis of many types of cancer, including OSCC [12,13,14,15,16,17]. Initially, cells with malignant transformation are identified and eliminated by representatives of the innate and adaptive immune system, preventing progression to cancer. This process can be disturbed, leading to clinically manifest tumors, through progressively acquired tumor cell resistance combined with a progressively altered antitumor immune response [18,19]. These complex tumorigenesis mechanisms involve a vast variety of cellular and non-cellular players. Among them, tumor-infiltrating lymphocytes (TILs) have been intensively studied in the quest to unveil the immune pathways involved in carcinogenesis. In OSCC, TILs influence tumor behavior, response to therapy and patient survival [20,21]. Different types of TILs play distinct, sometimes even opposing roles. The objective of our study is to investigate the immune landscape in OSCC, with a focus on CD4^+^, CD8^+^ and CD56^+^ cells. Immune cell distribution in different tumor compartments and correlations with clinicopathological features and their prognostic potential are in the scope of our investigation. Based on the density of TILs in different tumor areas, we aim to define prognostic risk groups in OSCC.

## 2. Materials and Methods

### 2.1. Patients and Tissue Samples

Patients with a histologically confirmed diagnosis of squamous cell carcinoma (SCC) were included in this study. All patients that were treated between 2016–2019 in the Department of Oral and Maxillofacial Surgery, “Carol Davila” Central Military Emergency Hospital Bucharest, were selected. The study was conducted in accordance with ethical guidelines, and received the approval of the Local Ethics Committee (No. 25/27.11.2017). The inclusion criteria for our study were: patients with confirmed diagnoses of SCC affecting oral and lip mucosa that did not receive any previous treatment and who were eligible for curative surgery. Patients with unresectable or metastatic tumors, with incomplete medical records or patients that were lost to follow-up were excluded. All patients underwent a thorough preoperative workup (clinical and imaging assessment) followed by radical resection of the tumor. Neck dissection was performed in all patients with positive nodes, as well as in patients with large tumors (T stages 3 and 4A) and clinically negative nodes. Surgery was followed by radiotherapy with or without chemotherapy in accordance with the national guidelines and patients entered a follow-up program, with visits that included clinical and imaging assessments.

### 2.2. Immunohistochemical Staining

The fragments harvested during the grossing of the specimens were routinely processed using a manual technique of histopathologic processing and paraffin embedded. Briefly, 3 μm sections were cut with a semi-automated Rotary Microtome Leica RM2245, on regular slides for routine and special stains, and on precoated slides for immunohistochemical tests. Immunohistochemical (IHC) tests were performed for CD4^+^ (Helper T lymphocytes), CD8^+^ (Cytotoxic T lymphocytes) and CD56^+^ (Natural Killer) cells. Specific details about clones, host, source, dilution and pretreatment are as follows: CD4 (clone 4B12 Leica), mouse, pretreatment with heat-induced epitope retrieval in EDTA, pH 8, dilution 1/200; CD8 (clone 4B11 Leica), mouse, pretreatment with heat-induced epitope retrieval in EDTA, pH 8, dilution 1/100; CD56 (clone CD564 Leica), mouse, pretreatment with heat-induced epitope retrieval in buffer citrate, pH 6, dilution 1/200. The detection system we used was Novolink Polymer (Leica/Novocastra) and DAB chromogen. Immunohistochemical stains were analyzed using an Olympus BX41 microscope.

### 2.3. Immunohistochemical Evaluation

**Figure 1:**
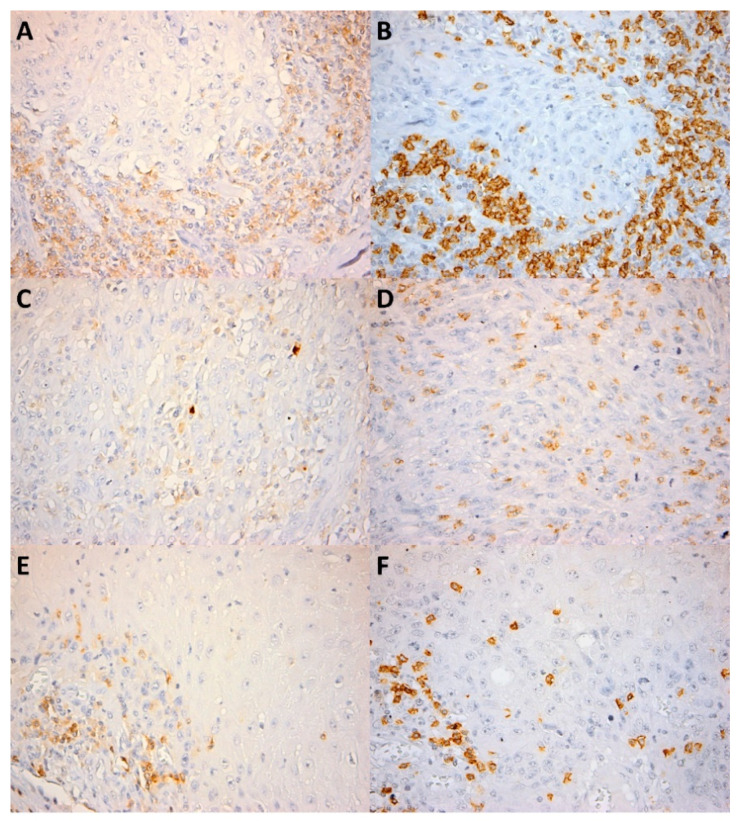
CD4^+^ and CD8^+^ lymphocytes in the intratumor compartment and at the invasion front. (**A**) Numerous CD4^+^ cells within the inflammatory infiltrate bordering the tumor invasion front, CD4 × 400; (**B**) Numerous CD8^+^ cells within the invasion front, CD8 × 400; (**C**) Few CD4^+^ cells within the intratumor inflammatory infiltrate, CD4 × 400; (**D**) Few CD8^+^ cells within the tumor, CD8 × 400; (**E**) Few CD4^+^ cells within the invasion front, CD4 × 400; (**F**) Few CD8^+^ cells within the invasion front, CD8 × 400. Note: Figure 1A and Figure 1B, Figure 1C and Figure 1D, Figure 1E and Figure 1F respectively, depict similar areas in the same tumor.

**Figure 2:**
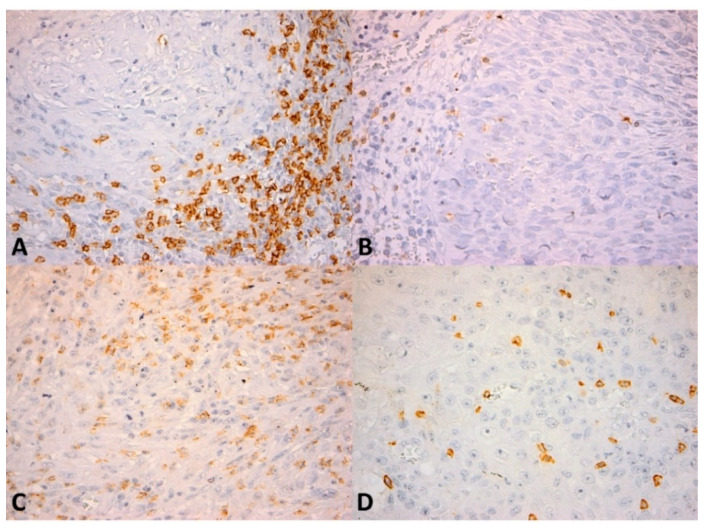
CD8^+^ lymphocytes in OSCC. (**A**) Numerous CD8^+^ cells (more than 106 cells/high power field) within the inflammatory infiltrate bordering the tumor invasion front; (**B**) Few CD8^+^ cells (less than 106 cells/high power field) within the invasion front; (**C**) Numerous CD8^+^ cells (more than 106 cells/high power field) within the intratumor inflammatory infiltrate; (**D**) Scattered isolated CD8^+^ cells (less than 24 cells/high power field) within the tumor. CD8 × 400.

**Figure 3:**
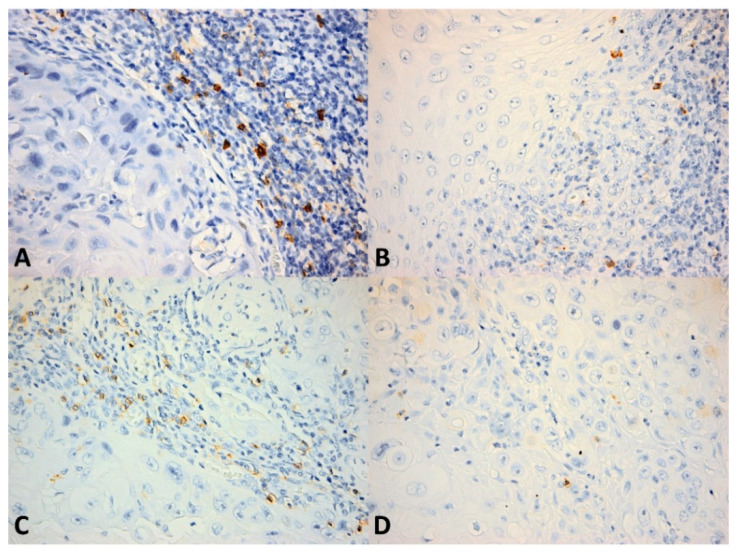
CD56^+^ cells in OSCC. (**A**) Numerous CD56^+^ cells within the inflammatory infiltrate bordering the tumor invasion front; (**B**) Few CD56^+^ cells (less than eight cells/high power field) within the invasion front; (**C**) Numerous CD56^+^ cells within the intratumor inflammatory infiltrate; (**D**) Scattered isolated CD56^+^ cells within the tumor. CD56 × 400.

We quantitatively evaluated the presence of CD4^+^ and CD8^+^ lymphocytes as the number of cells per high power field (HPF) (0.55 mm in diameter) both in the intratumor location and at the front of invasion (Figure 1 and Figure 2). The number of positive cells was appreciated in the hotspot by counting 10 adjacent HPFs. Also, the pattern of distribution was noted (absent, nodular, diffuse). CD56^+^ lymphocytes were evaluated as the number of cells per HPF, both intratumor and at the front of invasion (Figure 3). Due to the small number of positive cells (we recorded fewer than 20 CD56^+^ cells per HPF in all cases in the intratumor location and in all but seven cases at the front of invasion) it was not possible to evaluate the pattern of distribution.

### 2.4. Statistical Analysis

The data distribution within the study group was assessed using the Kolmogorov-Smirnov normality test. Data with parametric distribution were analyzed using Student’s *t*-test. Nonparametric distribution data were analyzed with the Wilcoxon-Mann-Whitney test. The correlations between clinicopathological parameters and each type of TIL were analyzed using Fisher’s exact test or the chi-square test. Prognostic data, defined as disease-specific survival (DSS), were calculated by the time interval from surgery to death caused by the disease. Overall survival (OS) was defined as the timeframe from surgery to last follow-up, for all living patients, or from surgery to death from any other cause, except the disease. Recurrence was defined as local, regional or metastatic disease. Receiver operating characteristic (ROC) analysis was performed for TILs that showed significantly different values between the DSS and OS groups. With the Youndex index, we determined cutoff points for each type of TIL. For survival analysis we used the Kaplan-Meier method, comparing results from a log-rank test for each group of patients. Univariate analysis and multivariate Firth penalized logistic regression analysis was subsequently performed. Statistical significance was considered to be *p* < 0.05. Statistical analysis was carried out with Prism 9 software (GraphPad) and SPSS version 23 (IBM).

## 3. Results

### 3.1. Patient and Tumor Characteristics

A total of 161 patients were confirmed with oral and lip SCC. Of these, 90 patients met the eligibility criteria, had sufficient tissue for analysis and were included in this study. The mean age of the patients was 63.34 years old (range 28–92). Most patients were males 70 (78%), and 20 (22%) were females. More than half of the patients confirmed a history of smoking and alcohol abuse (64% smokers and 50% confirmed alcohol consumption). Primary oral mucosa tumors, involving tongue, floor of the mouth, gingiva, palate and buccal mucosa, represented 60% of the study group. The remaining 40% were primary lip mucosa tumors extended or not to other oral mucosa sites. The mean follow-up time was 34 months, ranging from 18 to 48 months. In 26 (30%) patients, locoregional recurrence was recorded during the follow-up interval, and 20 (22%) patients died because of disease progression. Neck dissection, ipsilateral or bilateral, in tumors involving the midline, was performed in 47 (52%) patients. Histopathological confirmation of positive lymph nodes was reported in 26 (55%) patients, while the remaining 21 (45%) did not have node metastasis.

The analysis of correlations with clinicopathological features in our study group revealed that tumor size (defined as T stage) and TNM staging showed strong correlations with patient survival (*p* = 0.0009 for tumor size and *p* < 0.0001 for TNM staging). Oral tumors were associated with significantly more aggressive behavior compared to lip tumors (*p* = 0.0427). Positive margins after tumor resection were also associated with a worse prognosis (*p* = 0.0039). Local, regional or distant recurrence correlated with a poor patient prognosis (Table 1).

### 3.2. Assessment of Tumor-Infiltrating Lymphocytes

We have analyzed tumor-infiltrating lymphocytes (TILs) in tumor tissue (intratumor) and at the front of invasion. CD8^+^ and CD4^+^ lymphocytes showed major distribution differences between the two investigated compartments. High immune cell densities were seen at the front of invasion, with mean values of 159 CD8^+^ cells/HPF and 17 CD4^+^ cells/HPF, compared to the intratumor compartment where CD8^+^ lymphocytes had a mean value of 47 cells/HPF (*p* < 0.0001) and CD4^+^ lymphocytes had a mean value of two cells/HPF (*p* < 0.0001). CD56^+^ lymphocytes distribution did not follow the same pattern, with relatively similar densities in both the intratumor compartment and at the front of invasion. A diffuse pattern of distribution was seen in all patients, with significant immune infiltrate in both compartments (Table 2).

Analysis of TILs in the deceased after disease progression group, in contrast with the surviving group, revealed differences for specific immune cell subtypes in both compartments. Intratumor CD56^+^ lymphocyte density was significantly lower in the deceased group, with a mean value of five CD56^+^ cells/HPF, compared to 11 CD56^+^ cells/HPF in the surviving group (*p* = 0.0016). Similar results were found at the front of invasion, with higher densities of CD56^+^ cells in the surviving group. However, the reported values did not reach the threshold of statistical significance (*p* = 0.0622). Analysis of CD8^+^ lymphocyte infiltrate showed significantly lower densities of cells in the deceased group, in both compartments. At the front of invasion, CD8^+^ cells were almost twice as frequent in the surviving group compared to the deceased group, with mean values of 177 cells/HPF and 99 cells/HPF, respectively (*p* = 0.0011). Intratumor CD8^+^ infiltrate revealed higher densities in the surviving group, with mean values of 52 cells/HPF, compared to 29 cells/HPF in the deceased group (*p* = 0.0086). In our study group, we found no statistically significant differences in CD4^+^ lymphocyte infiltrate in the surviving patients compared to the deceased due to disease progression patients (Table 3).

### 3.3. ROC Curve Analysis and TILs Threshold Determination

**Figure 4:**
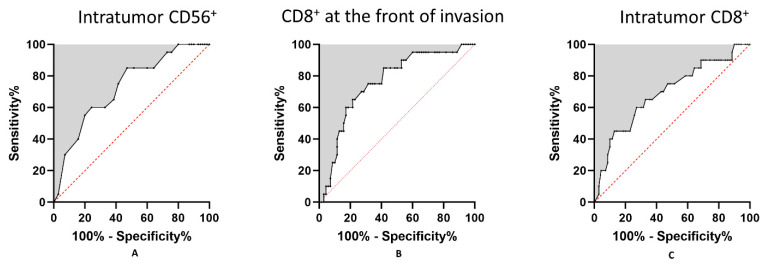
ROC analysis in OSCC. (**A**) Intratumor CD56^+^ cells; (**B**) CD8^+^ lymphocytes at the front of invasion; (**C**) Intratumor CD8^+^ lymphocytes.

Based on the above-reported data, cell types showing statistically significant differences between groups were selected for further analysis. We performed ROC curve analysis for CD56^+^ lymphocytes in the intratumor compartment and for CD8^+^ lymphocytes in the intratumor compartment and at the front of invasion. Results showed statistically significant differences for all three parameters. Intratumor CD56^+^ lymphocytes had an AUC of 0.7279 (*p* = 0.002), while CD8^+^ cells at the front of invasion resulted in an AUC of 0.7692 (*p* = 0.003). Intratumor CD8^+^ lymphocytes had an AUC of 0.6914 (*p* = 0.0093). Using the Youndex index, we determined cutoff values for each parameter as follows: for intratumor CD56^+^ lymphocytes, the value was eight cells/HPF; for CD8^+^ lymphocytes at the front of invasion, the value was 106 cells/HPF; for intratumor CD8^+^ lymphocytes, the value was 24 cells/HPF. Thus, two groups for each type of immune cell infiltrate were defined, high and low infiltrate groups, based on these cutoff values (Table 4 and Figure 4). 

### 3.4. Survival Analysis

**Figure 5:**
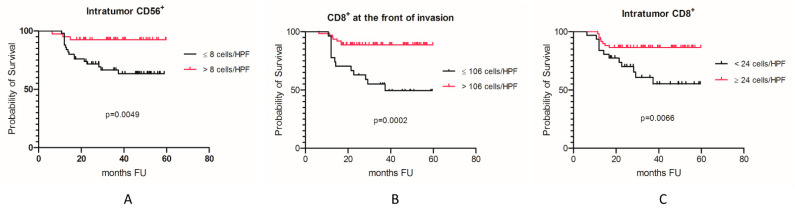
Kaplan-Meier curves in OSCC. (**A**) Intratumor CD56^+^ cells; (**B**) CD8^+^ lymphocytes at the front of invasion; (**C**) Intratumor CD8^+^ lymphocytes.

Survival analysis based on TILs revealed that intratumor CD56^+^ and CD8^+^ lymphocytes, as well as CD8^+^ lymphocytes at the front of invasion significantly correlated with patient prognosis (Table 5 and Figure 5). Intense immune infiltrates in the intratumor compartment with high values of CD56^+^ and CD8^+^ lymphocytes were positive prognostic factors in our study group (*p* = 0.0049 and *p* = 0.0066, respectively). Similar results were seen at the front of invasion for CD8^+^ lymphocytes, where high densities of CD8^+^ cells were correlated with a good prognosis (*p* = 0.0002).

### 3.5. Univariate and Multivariate Analysis of TILs and Clinicopathological Characteristics in OSCC

Univariate analysis of clinicopathological features in relation to the TILs categories was conducted. High densities of CD8^+^ lymphocytes at the front of invasion were found in most primary lip tumors (*p* = 0.0001) and in non-smoking patients (*p* = 0.0362). Patients with primary lip tumors also showed high intratumor CD8^+^ infiltration (*p* = 0.0061). Perineural invasion was frequently confirmed in patients with low intratumor CD8^+^ infiltration (*p* = 0.009). Similar to survival, locoregional recurrence correlated with all three analyzed parameters. Thus, high rates of recurrence were reported in patients with low intratumor CD8^+^ and CD56^+^ lymphocytes (*p* = 0.0262 and *p* = 0.0024) and low CD8^+^ infiltrate at the front of invasion (*p* = 0.0052) (Table 6).

Our multiple regression analysis pre-check showed that there was a complete separation in our data. We identified the responsible characteristic as being the presence of locoregional recurrence. According to these initial results, the presence of locoregional recurrence raised the risk of a patient belonging to the DSS group by 170.88 times (OR = 170.88, 95%CI 14.125–38,638.327, *p* < 0.001). Due to the complete separation of data upon running logistic regression analysis, Firth penalized logistic regression was used for this analysis, and locoregional recurrence was excluded from the analysis. The model was significant, with *p* < 0.0001. The results showed that if intratumor CD56^+^ was high (>8), the patient was 3.66 times more likely to belong to the surviving group (OR = 3.669, CI95% 1.09–15.37, *p* = 0.035) and if CD8^+^ at the front of invasion was high (>106), the patient was 3.75 times more likely to belong to the surviving group (OR = 3.75, CI95% 1.17–12.35, *p* =0.026). Thus, the predictive value of intratumor CD56^+^ infiltrate and CD8^+^ lymphocytes at the front of invasion were validated through multivariate analysis (multiple logistic regression–Firth method). In contrast, in multiple regression analysis, intratumor CD8^+^ infiltration did not hold predictive value (OR = 2.017, CI95% 0.15–1.62, *p* = 0.242).

When controlling for several clinicopathological parameters (age, sex, smoking status, tumor location, tumor degree of differentiation, T stage, TNM classification), none of these variables had statistically significant influences over the two aforementioned predictors. There were, however, three parameters approaching statistical significance: age (OR = 1.06, 95%CI 1–1.16, *p* = 0.063), moderately differentiated tumors (OR = 8.33, 95%CI 1.16–156.17, *p* = 0.07) and poorly differentiated tumors (OR = 11.97 95%CI 1.19–334.62, *p* = 0.069).

## 4. Discussion

The immune system plays a major role in tumor pathogenesis. In the quest for high fidelity prediction tools and, more recently, for individualized therapeutic targets, TILs have been studied as potential biomarkers in many types of cancer, including OSCC. In this study, we have investigated the characteristics of immune cell infiltrate in patients with OSCC and their prognostic potential. We found major differences in CD4^+^ and CD8^+^ lymphocyte density at the front of invasion compared to the intratumor compartment, with mean values three times higher for CD8^+^ lymphocytes at the front of invasion and eight times higher for CD4^+^ lymphocytes. In addition, intense infiltration with CD8^+^ lymphocytes in both compartments, intratumor and at the front of invasion, carried positive prognostic character. Patients belonging to the surviving group exhibited twice as many CD8^+^ lymphocytes in both compartments. These results showed consistency throughout statistical analysis only for CD8^+^ infiltration at the front of invasion. Our findings are in accordance with the results reported in another study that defined five distinct areas in OSCC tumors and reported a prognostic value only for CD8^+^ infiltration at the invading edge. The patterns of CD8^+^ cell distribution in different areas of the tumor were also concordant to our results, with four times more CD8^+^ cells present at the tumor periphery and invading edge compared to the central compartment [22]. Based on our findings, we identified a cutoff point and described two categories of tumors, with high and low CD8^+^ cell infiltration at the front of invasion. Patients that exceeded the threshold of 106 CD8^+^ cells/HPF at the front of invasion were less likely to relapse and die due to disease progression. This finding was not influenced by any of the clinicopathological features, thus supporting the independent character of CD8^+^ lymphocyte infiltrate at the front of invasion as a prognostic biomarker in OSCC. Similar findings have been reported in other studies that investigated the role of immune infiltration in different types of head and neck squamous cell carcinoma (HNSCC) [22,23,24]. Moreover, in other solid tumors, like colorectal, cervical or lung cancer, researchers reported similar results [25,26,27]. CD8^+^ cytotoxic lymphocytes, the main effectors of the adaptive immune response, are directly involved in tumor cell clearance and antitumor protection [28]. HNSCCs are considered highly immunogenic tumors, characterized by increased tumor mutational burden (TMB) and efficient tumor-specific antigen processing and presenting equipment that favors the local immune response [29]. TMB is influenced by exposure to the environmental factors, such as smoking and chronic UV radiation, involved in OSCC pathogenesis [30]. Higher infiltration with cytotoxic CD8^+^ lymphocytes was reported only in diffuse large B-cell lymphomas that have several folds more CD8^+^ cells compared to HNSCC, with a median of 1000 cells/HPF. The same study reported high values of CD8^+^ cells concentrated mainly at the invasive margin, in HNSCC, pancreatic and lung cancers [31]. In our group, tumors involving lip mucosa exhibited intense CD8^+^ lymphocyte infiltration in both compartments compared to other oral subsites, probably based on the same criteria of TMB, where—in addition to tobacco and alcohol abuse—chronic exposure to UV radiation was confirmed. This intense immunogenic behavior in lip SCC can contribute to the improved survival rates reported in patients with lip tumors [32]. Abundant tumor infiltration with CD8^+^ lymphocytes is not only a good prognosticator in most cancers, but also an argument for responsiveness to immune therapy [33]. Studies report that both CD8^+^ lymphocyte density and heterogeneity of distribution within tumor tissue are correlated with the response to immunotherapy [34]. Immune checkpoint inhibitors have been under intense investigation in many types of cancer, including HNSCC. This led to FDA approval in 2019 of anti-PD1 monoclonal antibodies, nivolumab and pembrolizumab, in advanced and metastatic HNSCC resistant to chemotherapy [35]. Exhausted immune cells overexpressing immune checkpoint molecules enter an anergic status with compromised functions, including an altered antitumor defense capacity [36]. In advanced HNSCC, overexpression of immune checkpoint inhibitors was found in immune enriched tumors, with a negative impact on patient survival [37]. However, in OSCC, a recent meta-analysis did not confirm their prognostic role [38]. Both CD4^+^ and CD8^+^ activated lymphocytes express programmed cell death protein-1 (PD-1) with inhibitory effects after binding to specific ligands present on tumor and immune cells [39].

If there is consistency in the literature regarding CD8^+^ lymphocyte tumor infiltration in most cancers, the results reported until now for CD4^+^ lymphocytes are far from being clarified. This might come from the diversity of CD4^+^ lymphocyte subtypes and their opposing functions. A systematic review on OSCC and OPSCC reported that most studies investigate the subset of regulatory T cells (CD4^+^Foxp3^+^) and despite narrowing the range of CD4^+^ cell types, the results are still contradictory, with a third of the studies associating an increased regulatory T cell infiltration with a poor prognosis, while the other two-thirds reporting improved outcomes in these patients [40]. In our study, we found a significantly higher infiltration with CD4^+^ cells at the front of invasion compared to the intratumor compartment. However, we did not find any significant relationship with the patient’s outcome in our study group. A meta-analysis of TILs in HNSCC reported that 10 out of 16 studies that have analyzed the prognostic role of CD4^+^ lymphocytes found no correlations with survival, five studies reported positive outcomes in patients with high CD4^+^ infiltration and one study reported a poor prognosis in these patients [41]. In his study, Spector found no prognostic correlations with CD4^+^ lymphocyte infiltrate in OSCC, but did report a decreased rate of death related to disease in the group of patients that underwent primary chemoradiation and displayed higher densities of CD4^+^ infiltrate [21].

Known as effectors of the innate immune system, natural killer CD56^+^ cells (NK) actively exert antitumor defense functions, as complementary players alongside the effectors of the adaptive immune response [42]. We have investigated NK cells in our study and, even though there were no differences in distribution between the front of invasion and intratumor compartment, with mean values of eight cells/HPF in both areas, we found that a higher intratumor infiltration with CD56^+^ cells significantly correlated with locoregional disease control and improved survival. A recent meta-analysis investigating NK cell population in HNSCC tumor tissue reported similar results [43]. Other studies have assessed NK cells in the peripheral blood of patients with OSCC, and found that NK cells are significantly decreased in patients compared to controls, but their number increases after tumor excision, displaying no differences after surgery compared to controls [44,45]. NK cells act as immune effectors independent of sensitization, in an HLA-free fashion. Tumor cell clearance is promoted in a nonspecific manner, through NK cell degranulation, cytokine release and cytotoxicity [46]. The signaling mechanisms for NK tumor infiltration can be explained by the so-called phenomenon of “missing self”, often seen in cells undergoing malignant transformation, which exhibit a phenotype lacking MHC class I molecules that engage the NK cells promoting tumor cell clearance [47]. The DNA damage accumulation reported in many environmentally-induced cancers, including nicotine and UV radiation-associated OSCC, activates NK cells by means of upregulated stress ligands [48]. Their effective nonspecific cytotoxic effects against malignant cells can justify their positive prognostic role in solid cancers [49]. Inhibitory effects of NK cells were detected in OSCC in peripheral blood and tumor tissue, with increased expression of suppressive cytokines interleukine-10 (IL-10) and tumor growth factor-β (TGF-β) and decreased expression of activating receptor NKp46 [50]. These recent findings open new perspectives in targeting the inhibitory signaling pathway, with consequent NK cell activation. Encouraging preliminary results have been reported in recurrent and metastatic NHSCC after administration of the immune checkpoint inhibitor targeting the NKG2A receptor expressed by NK cells in association with cetuximab [51]. Another promising research direction is focusing on engineered NK cells for cancer therapy. It has the advantage of general use, as opposed to T lymphocyte chimeric antigen receptor (CAR) therapies, which assume individualized therapeutic strategies, appealing in principle but challenging to implement in large populations of patients [46,52].

## 5. Conclusions

Our study confirms the key role of the innate and adaptive immune systems in OSCC. The predictive characteristics of CD8^+^ and CD56^+^ immune cells can be implemented as independent prognostic tools and can provide important elements in developing individualized therapy in the fight against OSCC and cancer in general.

## Figures and Tables

**Table 1:** Patient characteristics.

Variable				Survivors		Deceased		*p*-Value
		No	(%)	No	(%)	No	(%)	
		90			70	20		
Age, years (Mean ± SD)		63.34 ± 12.03						
Sex								0.5449
	Male	70	77.78	53	75.71%	17	85%	
	Female	20	22.22	17	24.29%	3	15%	
T stage								0.0009
	T1	18	20.00	17	24.29%	1	5%	
	T2	40	44.44	34	48.57%	6	30%	
	T3	17	18.89	13	18.57%	4	20%	
	T4	15	16.67	6	8.57%	9	45%	
Nodal status		47	52.22					0.0809
	pN0	21	44.68	19	27.14%	2	10%	
	pN^+^	26	55.32	17	24.29%	9	45%	
TNM stage								<0.0001
	I	16	17.78	16	22.86%	0	0%	
	II	28	31.11	27	38.57%	1	5%	
	III	18	20.00	13	18.57%	5	25%	
	IVA	28	31.11	14	20%	14	70%	
Location								0.0427
	Oral	54	60.00	38	54.29%	16	80%	
	Lip	36	40.00	32	45.71%	4	20%	
Smoking								0.0774
	Smokers	58	64.44	41	58.57%	17	85%	
	Nonsmokers	26	28.89	23	32.86%	3	15%	
	Missing	6	6.67	6	8.57%	0	0%	
Alcohol consumption								0.0895
	Drinkers	45	50.00	31	44.29%	14	70%	
	Nondrinkers	39	43.33	33	47.14%	6	30%	
	Missing	6	6.67	6	8.57%	0	0%	
Histological differentiation								0.2716
	High	19	21.11	17	24.29%	2	10%	
	Intermediate	56	62.22	43	61.43%	13	65%	
	Low	15	16.67	10	14.29%	5	25%	
Perineural invasion								0.1534
	Confirmed	13	14.44	8	11.43%	5	25%	
	Not confirmed	77	85.56	62	88.57%	15	75%	
Vascular invasion								0.6112
	Present	6	6.67	4	5.71%	2	10%	
	Absent	84	93.33	66	94.29%	18	90%	
Resection margins								0.0039
	Positive	12	13.33	5	7.14%	7	35%	
	Negative	78	86.67	65	92.86%	13	65%	
Locoregional recurrence								<0.0001
	Present	26	28.89	6	8.57%	20	100%	
	Absent	64	71.11	64	91.43%	0	0%	

Statistical significance <0.05.

**Table 2:** Distribution of tumor-infiltrating lymphocytes (TILs) in OSCC.

	Front of Invasion	Intratumor	*p*-Value
CD4^+^ lymphocytes	16.71	2.25	<0.0001
CD8^+^ lymphocytes	159.4	47.11	<0.0001
CD56^+^ lymphocytes	8.02	9.91	0.2717

Statistical significance <0.05.

**Table 3:** Tumor-infiltrating lymphocytes (TILs) in OSCC.

	Survivors		Deceased		*p*-Value
	Mean	SD	Mean	SD	
CD56^+^					
Front of invasion	8.486	4.883	6.4	4.903	0.0622 ^
Intratumor	11.37	17.17	4.8	4.238	0.0016 ^
CD8^+^					
Front of invasion	176.7	97.04	98.7	66.5	0.0011 *
Intratumor	52.24	38.85	29.15	30.74	0.0086 ^
CD4^+^					
Front of invasion	16.55	19.27	17.3	28.21	0.4128 ^
Intratumor	2.543	4.01	1.25	1.552	0.3093 ^

Cells/HPF; SD = standard deviation; * *t*-test; ^—Mann-Whitney test.

**Table 4:** ROC analysis.

		CD56^+^ Intratumor	CD8^+^ Front of Invasion	CD8^+^ Intratumor
Area under curve		0.7279	0.7582	0.6914
Std. error		0.06154	0.05871	0.06952
95% CI		0.6072 to 0.8485	0.6431 to 0.8733	0.5552 to 0.8277
*p* value		0.002	0.0005	0.0093
Cut-off	Low	≤8	≤106	<24
	High	>8	>106	≥24
Sensitivity%		85	65	60
Specificity%		52.86	78.57	72.86

Statistical significance <0.05.

**Table 5:** Survival analysis.

		Survival	Log-Rank Test	*p*-Value
CD56^+^ intratumoral			7.912	0.0049
	high > 8	92.50%		
	low ≤ 8	66.00%		
CD8^+^ front of invasion			13.67	0.0002
	high > 106	88.71%		
	low ≤ 106	53.57%		
CD8^+^ intratumoral			7.378	0.0066
	high ≥ 24	86.44%		
	low < 24	61.29%		

Statistical significance <0.05.

**Table 6:** Correlation analysis with clinicopathological features in OSCC.

Variable		CD56^+^ Intratumor		*p*-Value	CD8^+^ Front of Invasion		*p*-Value	CD8^+^ Intratumor		*p*-Value
		≤8	>8		≤106	>106		<24	≥24	
Sex				0.7997			0.2811			0.7912
	Male	38	32		24	46		25	45	
	Female	12	8		4	16		6	14	
T stage				0.0541			0.0818			0.3276
	T1	8	10		3	15		6	12	
	T2	18	22		10	30		11	29	
	T3	13	4		7	10		9	8	
	T4	11	4		8	7		5	10	
Nodal status *				0.7463			0.2316			0.2451
	pN0	16	5		6	15		6	15	
	pN+	18	8		13	13		12	14	
TNM stage				0.0713			0.0606			0.1363
	I	7	9		2	14		4	12	
	II	12	16		6	22		6	22	
	III	10	8		7	11		9	9	
	IVA	21	37		13	15		12	16	
Location				0.0898			0.0001			0.0061
	Oral	34	20		25	29		25	29	
	Lip	16	20		3	33		6	30	
Smoking				0.0956			0.0362			0.1684
	Smokers	32	19		23	35		24	34	
	Non-smokers	17	26		3	23		6	20	
	Missing	1	5		2	4		1	5	
Acohol consumption				0.1388			0.1559			0.4299
	Drinkers	26	19		18	27		18	27	
	Non-drinkers	23	16		8	31		12	27	
	Missing	1	5		2	4		1	5	
Histological differentiation				0.6690			0.8754			0.5732
	High	9	10		5	14		8	11	
	Intermediate	33	23		18	38		17	39	
	Low	8	7		5	10		6	9	
Perineural invasion				0.7667			0.1011			0.009
	Confirmed	8	3		7	6		9	4	
	Not confirmed	42	35		21	6		22	55	
Locoregional recurrence				0.0024			0.0052			0.0262
	Present	21	5		14	12		14	12	
	Absent	29	35		14	50		17	47	

* from a total of 47 neck dissections.

## Data Availability

The datasets used and/or analyzed during the present study are available from the corresponding author.
